# Quality of life of children treated for cleft lip or palate in a selected South African population: a questionnaire-based survey of guardian/parent perspectives

**DOI:** 10.1007/s10006-025-01466-9

**Published:** 2025-10-15

**Authors:** Siyabonga Lembede, Okikioluwa Stephen Aladeyelu, Anil Madaree, Lelika Lazarus

**Affiliations:** 1 Department of Clinical Anatomy, School of Medicine, University of KwaZulu-Natal, Durban, South Africa; 2 Faculty of Medicine and Health Sciences, Walter Sisulu University, Mthatha, South Africa; 3 Department of Plastic and Reconstructive Surgery, Inkosi Albert Luthuli Central Hospital, Durban, South Africa

**Keywords:** Cleft lip, Palate, Quality-of-life, Physical, Psychological, Social health

## Abstract

**Background:**

Cleft lip or palate are defects that affect the orofacial region and continue to be a serious public health challenge impacting the quality of life of affected patients. Surgical repair of the cleft lip or palate has been the most effective treatment for correcting these facial defects. This study aimed to evaluate the quality of life of children treated for cleft lip or palate in a selected South African population.

**Methods:**

Fifty patients aged 1 to 13 years, diagnosed with non-syndromic cleft lip or palate, were evaluated six months post-surgery. The QoL of children with cleft lip and/or palate was assessed through parent-reported Likert scale ratings across physical, psychological, and social health domains.

**Results:**

Patients with cleft lip only reported lower QoL scores in the social health domain (63.5%). Those with unilateral cleft lip and palate showed reduced scores in the psychological health domain (57.1%), while patients with bilateral cleft lip and palate had lower scores in the physical health domain (71.1%). In contrast, children with cleft palate only demonstrated higher QoL scores across all health domains. Female participants reported higher scores in both the psychological (61.5%) and physical (85%) health domains. Notably, children aged 10–13 years had the lowest scores across all domains of health.

**Conclusion:**

Patients with a cleft palate only showed an increase in QoL than the other cleft subtypes. Females had an increased QoL than males, and children in the 1-3 age group exhibited higher QoL scores than older age groups.

## Introduction

Cleft lip and palate are the most common congenital malformations of the head and neck, observed on the lips, hard and soft palates [1]. Most African countries lack active population-based surveillance programs for the screening of clefts. Prevalence is determined by using hospital-based data [2]. Only a few studies have been done in South Africa to ascertain the epidemiology of clefts [3]. A prevalence rate of 0.3 per 1000 live births was reported in South Africa [4]. Cleft lip or palate are linked to several complications that impact patients’ quality of life (QoL) in the physical, functional, and psychological domains, compromising their ability to eat, communicate, hear, and maintain their physical appearance [5]. These factors affect patients’ well-being and self-esteem, which in turn have a direct impact on their social interactions and relationships [6]. Individuals born with clefts ideally receive multidisciplinary care that includes several surgical procedures, hospital stays, and repeated follow-up appointments throughout their childhood [6].

In 1947, the World Health Organization (WHO) first described QoL as a “state of complete physical, mental, and social well-being, and not merely the absence of disease and infirmity [7]. The WHO's definition has expanded over time to encapsulate an individual's perspective of their position in life as situated within the cultural and ethical frameworks that form their world regarding their goals, expectations, standards, and concerns [7]. It is a broad concept that encompasses the complex integration of an individual’s physical health, psychological state, level of independence, social connections, personal beliefs, and their relationships to key aspects of their environment [8].

Children with cleft lip or palate experience significant challenges associated with being visibly different, having speech and language difficulties, and social isolation [9]. An individual's psychological well-being is highly influenced by their perception of their facial attractiveness and their ability to communicate verbally [10]. Orofacial clefts may also often be linked to severe clinical and psychological consequences like anxiety, depression, and low self-esteem [11]. As a result, their management will significantly impact the patient's QoL and general well-being [11]. Cleft care in developing nations demonstrably lags behind that of high-income, well-resourced countries, primarily due to significant constraints such as limited financial resources, inadequate healthcare infrastructure, and critical shortages in the specialized workforce required for comprehensive cleft management [11].

Through various studies, social science research has continued to point out a strong bond between body appearance, stereotypes that people start to form, and the expectations that are consequently cultivated [12]. Social media platforms are widely accessible and significantly influence users' decision-making processes concerning various life domains, including health-related behaviors. However, most social media platforms create a serious risk of encountering misinformation [13]. Knowledge of this, in essence, cyclically flows back to demonstrate the extent to which appearance plays in shaping social perception and expectations of what certain physical looks or features signify [12]. Patients with clefts reportedly experience rates of anxiety and depression that are twice as high as those of the general population [12]. Depression in individuals with clefts has been associated with dissatisfaction regarding their appearance. The literature further suggests that various aspects of social functioning— including appearance-related satisfaction, anxiety, depressive symptoms, and family dynamics—may be adversely impacted [12].

Having the patients report their own perception of their QoL is very helpful in recognizing which measures can be taken and in what ways treating and caring should be improved; otherwise, the treatments may be those through which the patients derive just minimal benefits [14]. QoL is used to highlight the different hardships that can affect individuals. If patients are given this information, they will be able to anticipate and understand the outcomes of the deformity and its treatment [14, 15]. Difficulties can persist in individuals born with clefts even after treatment. These late problems may not be detected without a QoL assessment. Since QoL is both a major prognostic factor and a predictor of response to treatment, good or bad, it will also become crucial for making medical decisions [14].

Treating cleft lip or palate is a comprehensive process that starts at birth and extends into adolescence and adulthood. Maxillofacial surgeons routinely perform surgical reconstruction for clefts, addressing lip repair around 4–6 months of age and palate repair between 8 to 14 months, and beyond, while clinical psychologists focus on psychological rehabilitation [16, 17]. Ongoing care involves regular clinic visits to manage health issues such as mid-facial growth deficiency, hearing challenges, ear infections, speech impairments, dental anomalies, and alveolar bone defects [17, 18]. The prolonged nature of these multidisciplinary treatments places a considerable psycho-emotional and financial burden on both the patient and their family, often impacting social relationships and coping mechanisms [17]. Accordingly, a questionnaire for individuals with cleft lip or palate could be able to address areas of importance and be aware of any changes in QoL aspects as the child matures. The current research, therefore, aimed to determine the effect of surgical repair of cleft lip or palate on the QoL in patients with orofacial clefts in a South African population, according to types of clefts, age, and sex.

## Materials and methods

### Study design

This is a cross-sectional study done at a Central Hospital in Durban, South Africa, with 50 patients between the ages of 1 to 13 years suffering from non-syndromic cleft lip or palate who underwent primary or secondary surgical repair within six months prior to the study.

### Ethical approval

Institutional ethical approval for this study was obtained from the Biomedical Research Ethics Committee (BREC/00004708/2022) at the University of KwaZulu-Natal. Informed and written consent was obtained from the participants and their guardians.

### Consent

As the participants in this study were younger than 18 years of age, an assent form was provided to their parent/guardian, after which an informed consent form was also requested to be completed.

### Sample size determination

Using G-power software, the following statistical parameters were used to arrive at a minimum sample size with a statistical power of 90% with an effect size equal to 0.49. Type 1(Alpha) error equal to 0.05 (this is the false positive), Type 2 (Beta) error equal to 0.1 (this is the false negative), and statistical power = 1-β = 0.9 (which means a statistical power of 90%). Based on the above statistical parameter, a minimum sample size of 36 patients was determined; however, 50 patients were used to increase the statistical power of the study.

### Data collection tool

**Fig. 1:**
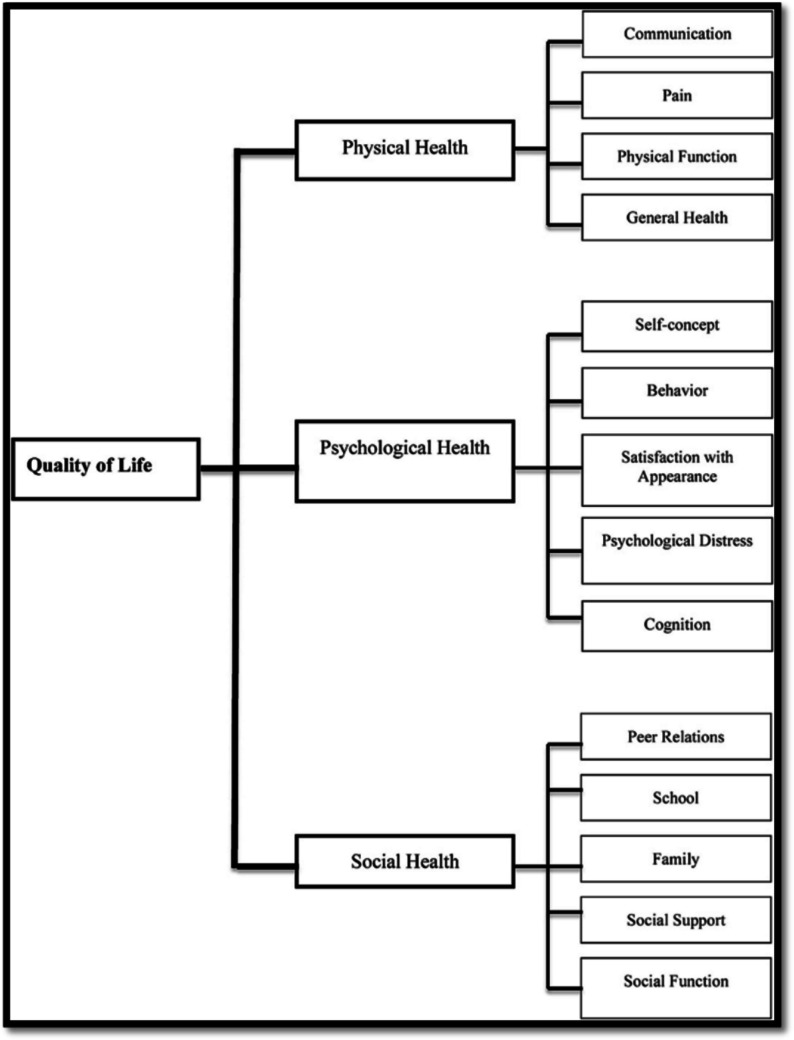
QoL health domains (Adapted from Klassen et al., 2012)

A structured questionnaire adapted from Menon et al. [19] was utilized for this study. This questionnaire was adapted because it is more disease-related, specifically to cleft lip/palate, as used by Menon et al. [19] in the South Indian population. The questionnaire was structured into four sections, covering socio-demographic information, physical health, psychological health, and social health, following the conceptual framework developed by Klassen et al. [20] (Fig. 1).

Section A was the socio-demographic information section, which included particulars of participants like age, sex, type of cleft, and side of cleft. Section B was the physical health section, and it comprised items on physical functioning, such as feeding, breathing, speech, etc. Section C was the psychological health component and included satisfaction with appearance, psychological distress, and self-concept items. Section D focused on the social health component. It consists of items on social functioning and social support, including peer and family relations. Sections B, C, and D included a four-point Likert scale on which participants were asked to rate how frequently these events occurred, where:

- 4 = No
- 3 = Do not know
- 2 = sometimes
- 1 = Yes

### Validity and reliability of questionnaires

The questionnaire was drafted in consultation with a biostatistician and subsequently presented to the supervisors for face and content validation. The questionnaire was validated using the Cronbach’s alpha reliability analysis test. Cronbach's Alpha is a measure of reliability, or internal consistency. It evaluates the consistency of surveys using multiple-question Likert scales, which assess underlying variables such as an individual's awareness, feelings of anxiousness, or honesty. Cronbach's Alpha ranges from 0 to 1, where a higher value reflects greater internal consistency. Generally, a value of 0.70 or higher is considered acceptable for research. The Cronbach’s Alpha value was calculated to be above 0.72 for all three health domains. A pilot study was carried out using 20% of the total sample size intended for the main research to check for the reliability of the questionnaire.

### Method of data collection

Upon arrival at the Craniofacial Clinic, the principal investigator verbally explained the study details to each participant. Subsequently, each participant received an information sheet, and those who agreed were enrolled consecutively. As all participants were under 18 years old, parents or guardians provided responses on their behalf. When necessary, the principal investigator explained certain terms in one of South Africa's local languages. Following respondents' consent, the interviews were documented. The principal investigator conducted all interviews individually, and no follow-up interviews were required for any participant. On average, each interview lasted approximately 10 min.

### Statistical analysis

Statistical data analysis was conducted using the R Statistical computing software of the R Core Team, 2020, version 3.6.3. To assess differences in QoL scores, participants were categorized into four subgroups: bilateral cleft lip and palate, unilateral cleft lip and palate, cleft lip only, and cleft palate only. SAS statistical software was employed to compile demographic data such as cleft type, laterality, age, and sex. Analysis of Variance (ANOVA) was employed to compare QoL scores across different cleft subtypes. The Chi-square test of independence was used to examine associations between QoL scores and sex. Additionally, ANOVA was applied to analyze QoL scores across different age groups. QoL scores were calculated by summing up all the scores for each patient; high scores on each health domain imply a higher QoL.

#### **Determination of QoL**

The Median-Based Cut-off method was used, and a benchmark of 50.5% was considered; scores falling below or close to this mark represented low QoL, while those that exceeded this number represented high QoL.

## Results

### Demographics

A total number of 50 participants were included in this study. The median age of the participants was 3.00, with an interquartile range of 2.00 to 3.00 (Table 1).

**Table 1:** Participants' demographics

Type of Cleft *n* (%)	Laterality *n* (%)	Age	Sex *n* (%)	Ethnicity *n* (%)	Attending regular/special school *n* (%)
Bilateral lip; Palate 4 (8.0)	Left 18 (36.0)	Median (Q1-Q3)	Female 25 (50.0)	African 40 (80.0)	Not applicable 26 (52.0)
Lip 8 (16.0)	Left & Right 3 (6.0)	3.00 (2.00–7.00.00.00)	Male 25 (50.0)	Asian 9 (18)	Regular 21 (42.0)
Lip & Palate 25 (50.0)	Middle 10 (20.0)	n (Min-Max)		Caucasian 1 (2.0)	Special 3 (6.0)
Palate 13 (26.0)	Right 19 (38.0)	50 (1.00–13.0.00.0)			

### Physical health domain

**Fig. 2:**
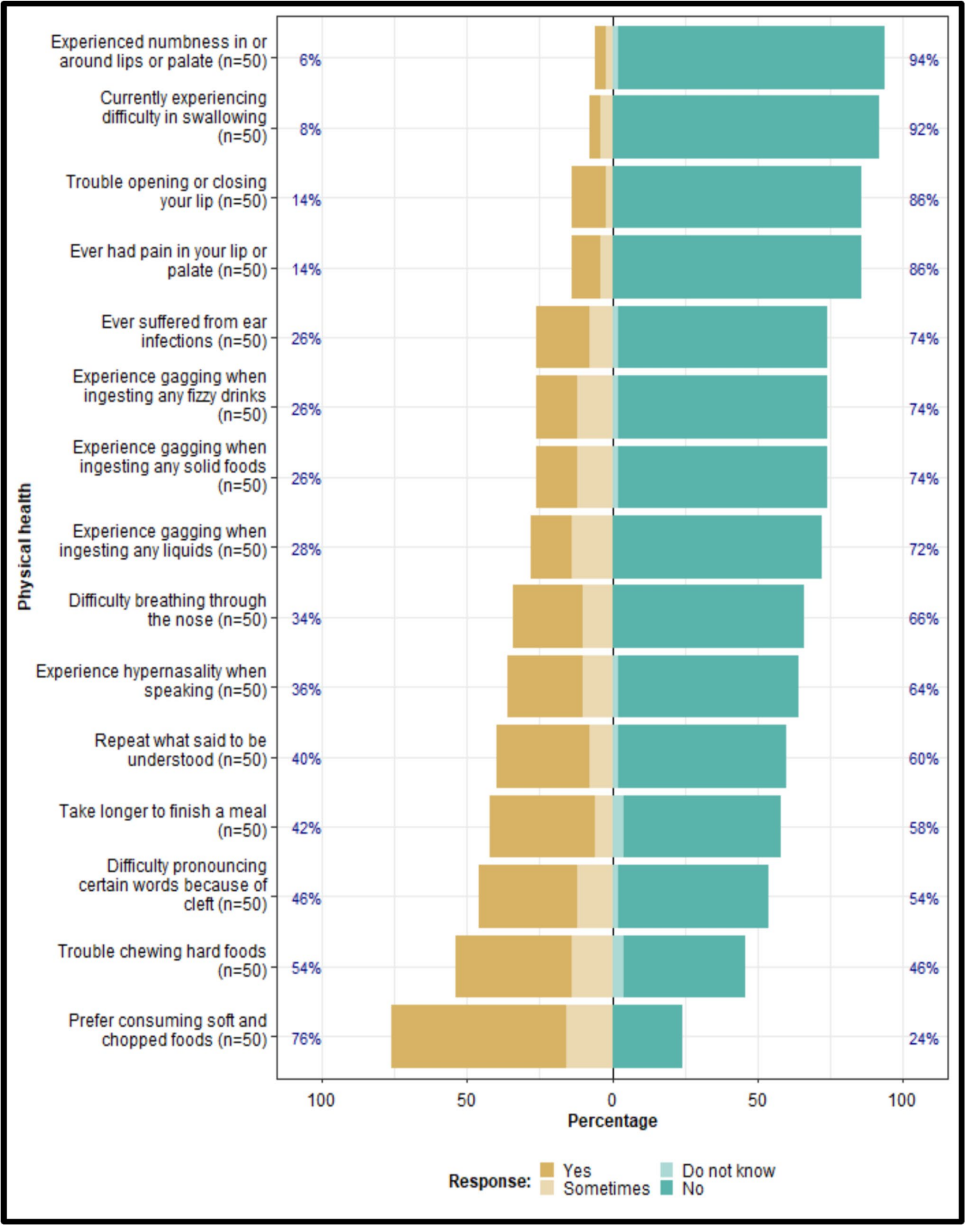
Physical health domain

The majority of the participants’ guardians (76%) reported that their children experienced feeding difficulties and a preference for soft or chopped foods. Only a small proportion (8%) experienced swallowing difficulties. Over 30% reported having difficulties breathing through the nose and hypernasality during speech, while more than 40% reportedly had challenges pronouncing certain words, which necessitated repeating themselves to be understood (Fig. 2).

### Psychological health domain

**Fig. 3:**
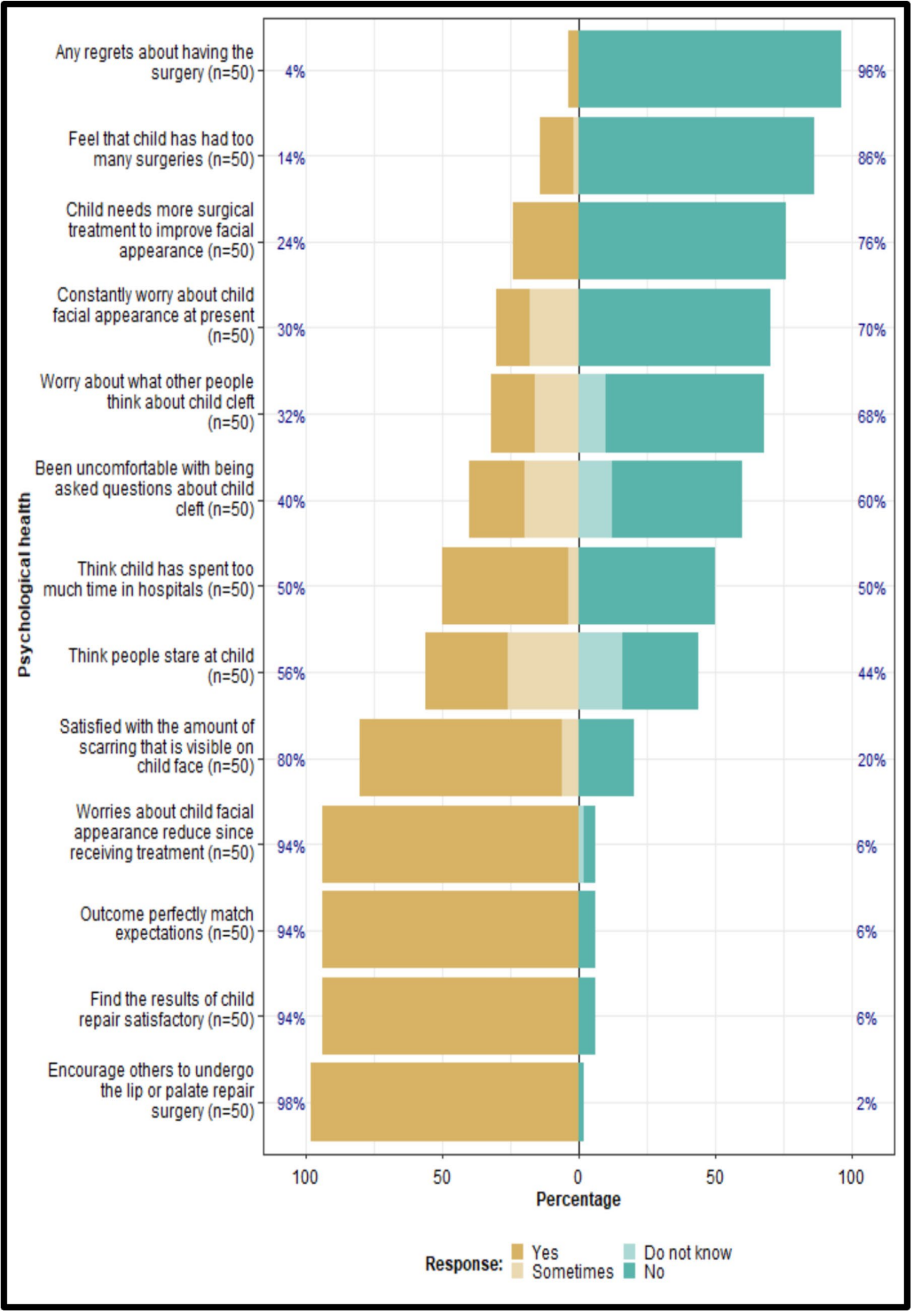
Psychological health domain

Over 90% of parents reported satisfaction with the surgical outcomes, indicating that the results met their expectations and that they would recommend lip or palate repair surgery to others. Half of the respondents indicated that they had spent an excessive amount of time in hospitals, with 14% reporting that their children had undergone an excessive number of surgeries and 24% expressing a desire for additional surgical interventions. Only 4% of parents expressed regret regarding the decision to proceed with the procedure (Fig. 3).

### Social health domain

**Fig. 4:**
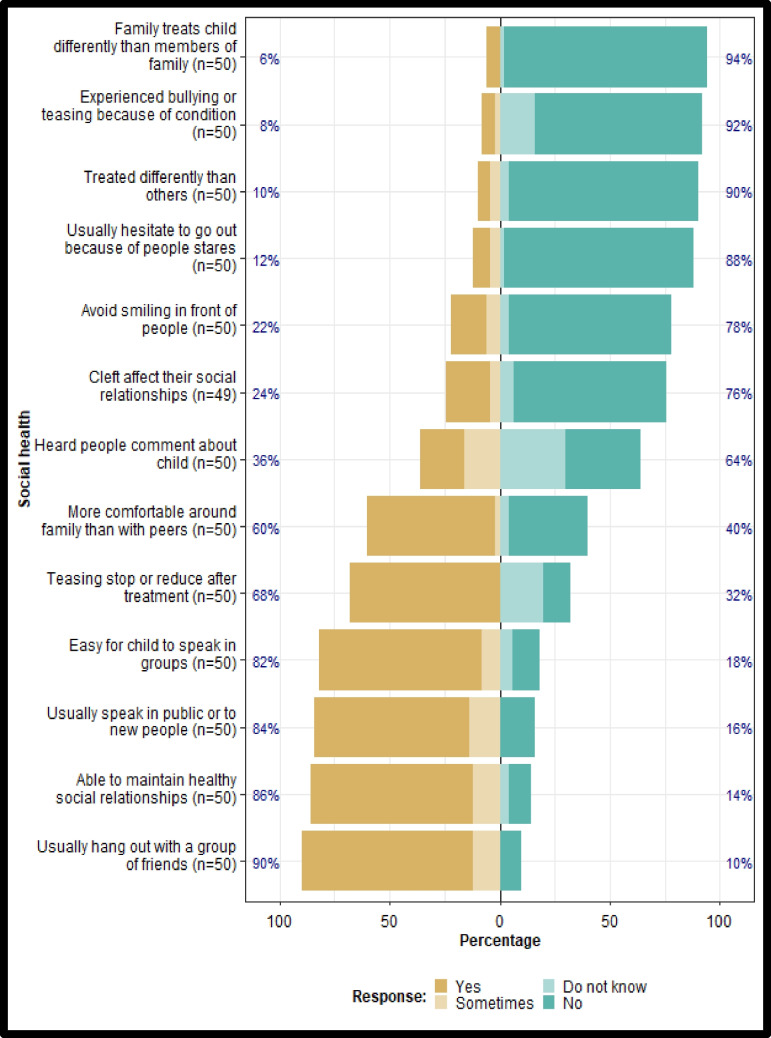
Social health domain QoL Scores

More than 80% of guardians reported that their child found it easy to speak in groups, with new people, or in public settings, suggesting the ability to maintain healthy social relationships. Ten percent perceived that their child was treated differently from others, while 8% reported experiences of bullying or teasing. Over 20% indicated that their child avoided smiling in public, 36% noted that others made comments about their child, and 12% stated that their child hesitated to go out due to being stared at by others (Fig. 4).

### QoL outcomes

**Fig. 5:**
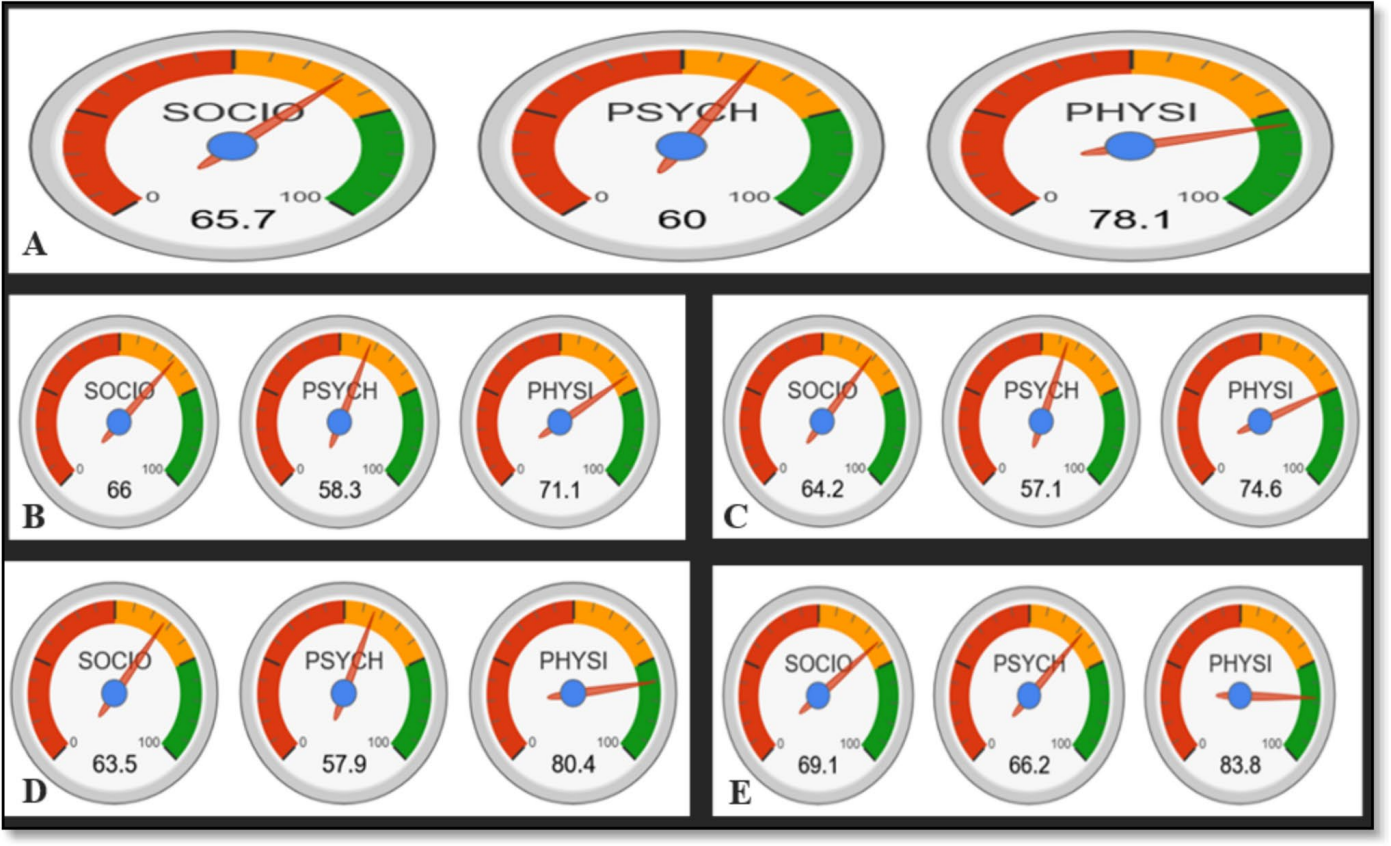
QoL scores for all types of clefts: **A**) overall QoL scores for all types of clefts, **B**) bilateral cleft lip and palate, **C**) unilateral cleft lip and palate, **D**) cleft lip only, **E**) cleft palate only

Overall, physical health QoL scores were uniformly high across all cleft types, with values exceeding 70.0, while psychological health QoL scores were relatively lower for each cleft type, approximating values near 50.0 (Fig. 5).

Social scores were high for individuals with cleft palate only and low for those with cleft lip only; however, this difference was not statistically significant (*p* = 0.218). A statistically significant difference was observed between participants with cleft lip only and those with cleft palate only, where psychological scores were low for the cleft lip group and high for the cleft palate group (*p* < 0.05). Physical scores were low for participants with unilateral cleft lip and palate, bilateral cleft lip and palate, and cleft lip only, while those with cleft palate had higher scores; however, no statistically significant differences were observed (*p* = 0.173) (Table 2).

**Table 2:** QoL scores of patients post-surgery categorized by the type of cleft

TYPE OF CLEFT	UCLP	BCLP	CL	CP	*P-*VALUE
PHYSICAL SCORE	74.6	71.1	80.4	83.8	0.173
SOCIAL SCORE	64.2	66.0	63.5	69.1	0.218
PSYCHOLOGICAL SCORE	57.1	58.3	57.9	66.2	0.004*
OVERALL QoL	65.3	65.13	67.27	73.0	0.273

*UCLP*, Unilateral cleft lip and palate; *BCLP*, Bilateral cleft lip and palate; *CL*, Cleft lip; *CP* – Cleft palate. *p < 0.05 is significant

No statistical significance was detected in social scores between males and females, as the values were equal (*p* = 0.863) (Table 3). Psychological scores were slightly higher in females than in males, but this difference was not significant (*p* = 0.415). Physical scores were higher in females compared to males, but this difference was not significant (*p* = 0.534).

**Table 3:** QoL scores of patients post-surgery categorized by sex

SEX	FEMALE	MALE	*P-*VALUE
PHYSICAL SCORE	85.0	78.3	0.287
SOCIAL SCORE	65.4	65.4	0.892
PSYCHOLOGICAL SCORE	61.5	59.6	0.415
OVERALL QoL	70.6	67.8	0.123

There is a decrease in the social score with increasing age across age groups; however, this decrease was not significant. (*p* = 0.380) (Table 4).

**Table 4:** QoL scores of patients post-surgery categorized by age

AGE GROUPS	1–3	4–6	7–9	10–13	*P-*VALUE
PHYSICAL SCORE	81.3	26.2	25.5	23.1	0.398
SOCIAL SCORE	67.0	23.0	22.4	19.7	0.380
PSYCHOLOGICAL SCORE	59.4	20.7	20.3	20.3	0.416
OVERAL QoL	69.2	23.33	22.7	21.0	0.393

## Discussion

The concept of QoL has won the attention of academics and practitioners alike and is now recognized as an essential domain of study and practice in the field of health and medicine [14]. QoL is integral for symptom relief, care delivery, and patient reintegration [15]. In the last few decades, significant advancements in medical care, including technological innovations, new medications, and enhanced understanding of disease mechanisms, have led to a substantial increase in the average life expectancy of patients [21]. However, improvements in life expectancy do not always translate into improvements in QoL. Thus, it is imperative that all medical professionals put patients' QoL first [22].

### Physical health domain

Children with this condition face a variety of functional and aesthetic challenges. These challenges include feeding complications since birth due to issues with oral sealing, swallowing, nasal regurgitation, and hearing problems due to abnormalities in the musculature of the palate, as well as speech defects due to problems in nasal escape, articulation, and dental matters [23]. While 76% of the participants reported that they preferred consuming chopped foods even after surgery, only 8% stated that they currently experience difficulties when swallowing, and 26% still experienced regurgitation. A notable difference was reported between males and females in this domain, with females reporting more improvements to their QoL as opposed to males. However, this difference was not statistically significant (*p* = 0.287).

### Social health domain

Professionals caring for patients with clefts indicate that many individuals experience social effects due to the condition [24]. In a qualitative study by Tiemens et al. [25], participants indicated that having a cleft lip resulted in increased shyness and reduced confidence [25]. In this study, only 12% of the participants reported that they avoided going out because people stared at them. Using social isolation and distraction as methods for coping led to poor social experiences and poor adjustment [18]. According to previous studies, children with cleft lip or palate have a greater percentage of school dropouts and are less likely to participate in clubs and groups than their peers [26, 27]. The results of the present study differ in that 84% of participants reported that they usually speak in public or in their respective groups. This was supported by 8% of the participants who reported that they had been bullied. There was no notable difference between males and females in this domain, as both groups showed similar improvements in their QoL (*p* = 0.892).

### Psychological health domain

Anxiety and depression have been reported to be twice as prevalent among individuals with clefts compared to normal control patients [28]. Dissatisfaction with facial appearance has been found to be a predictor of depression among subjects with cleft [29]. According to a study by Noor and Musa [30], many patients stated that the cleft had influenced or "greatly impacted" their self-confidence [30]. In the present study, although over half of the participants stated they had spent too much time in hospitals and thought people stared at them, the overall perception showed that most (94%) were very satisfied with the outcome of their repair surgery. Also, their concerns about their facial appearance decreased after treatment. This is consistent with other studies in which children with cleft lip or palate achieved higher scores in terms of self-image compared to control patients [10, 31]. While it is common in other studies that females with cleft lip or palate are more dissatisfied with their appearance than males [29, 32, 33], females in this study were more satisfied with the amount of scarring visible on their faces, which led to a relatively higher QoL than males.

### Overall QoL

Efforts have been undertaken to measure the impact of dental and oral disorders on daily life and overall well-being, as well as their influence on clinical care outcomes, such as the effectiveness of treatment interventions [34]. Patients with cleft lip or palate are very conscious of how others perceive them because their condition may be hard to conceal and could evoke negative feelings.

Considering the nature of the cleft, Payer et al. [35] reported that patients with cleft lip only showed the highest QoL score, followed by those with cleft palate only, those with a unilateral cleft lip and palate, and lastly those with a bilateral cleft lip and palate [35]. Similar studies done by Kramer et al. [36] and Sahoo [37] had the same results, with patients who were treated for cleft lip only having the highest QoL scores, followed by the cleft palate only group, and lastly, the unilateral cleft lip palate group [36, 37]. A study conducted by Bos et al. [32] also reported that patients treated for cleft lip only had high QoL scores. However, this group was followed by those with bilateral cleft lip and palate, unilateral cleft lip and palate, and cleft palate only [32]. Unlike the previous studies, results of this present study revealed minor differences in patients treated for cleft palate only, scoring the highest on QoL, followed by patients with cleft lip only, then unilateral cleft lip and palate, and lastly, patients with bilateral cleft lip and palate. It is necessary to note that different types of clefts require different treatment methods. These facts may clarify, at least partially, the differences reported.

Furthermore, regarding sex, previous studies report that female cleft patients are less satisfied with their facial appearance and have lower health-related QoL when compared with their male counterparts [29, 33–38]. Defabianis et al. [5] found QoL scores to be higher for males than females in an Italian population [5]. These findings are comparable to those of Bos et al. [32], who evaluated the quality of life of males and females and found males to have higher scores than females [32]. This observation is supported by the fact that females usually worry more about their body image and are more susceptible to the stigma associated with cleft repair than males [39]. This contradicts the results of the present study, which found females to have higher scores than males; however, it agrees with Eslami et al. [40], who examined the oral health-related QoL among Iranian patients and reported that females scored higher than males [40], these differences could also be due to the small sample size and the fact that surgical procedures required for each patient with cleft lip and palate will vary depending upon the type and severity of the deformity. In a similar study, Nolte et al. [41] provided an overview of QoL sex differences and indicated that females scored higher than males, which is similar to the works of previous authors [35, 36, 41, 42].

Some attention has been paid to variation with age. According to a review by Hunt et al. [10], age does not seem to have any significant influence on the QoL of cleft participants [42]. However, there are some exceptions to this, with increasing reports of more challenges as the child grows. This can be seen in the decreasing QoL scores of participants over the age of 12. Previous authors have compared QoL of children in the age groups 8–12 and 12–15 and have found the latter to have significantly lower scores [5, 32, 35]. The present study had similar results, with participants in the 1–3 age group having higher QoL scores than the 10–13 age group. The observed variations may be attributed to the limited capacity of young children to accurately self-report due to cognitive and communication challenges, whereas patients aged 12 and above typically demonstrate greater self-reflection, observational skills, and improved ability to communicate their concerns to their parents. This developmental difference likely explains the age-related disparities identified in the present study. An additional consideration is that the participants in this study were treated by different surgical teams, which may produce variable results.

## Conclusion

This study evaluated whether QoL increases in children with cleft lip or palate after surgical treatment. Although with a limited sample size of fifty, the present study reported high QoL scores among patients affected by cleft lip and palate. However, even after surgery, some domains of physical, psychological, and social health remain affected. Patients affected by bilateral and unilateral cleft lip and palate have considerably lower QoL scores compared to individuals affected by cleft palate only. Females were less affected by the clefts, as their QoL scores were higher than those of males. Additionally, participants aged 1 to 3 reported the highest quality of life scores when compared to those in older age groups.

### Limitation

One of the limitations of this study is the absence of a control group, which could have allowed for comparison with QoL scores of unaffected patients. Another limitation to consider is that cleft lip and palate repair can be performed using a variety of procedures. Also, every surgeon incorporates their own modification to make it a variation.

### Recommendation

Further investigations with larger sample sizes for each cleft type, along with the inclusion of a control population, are necessary to produce more robust and convincing results. This approach would facilitate meaningful comparisons across different cleft types, populations, and age groups. To improve the assessment for patients with orofacial clefts, it is crucial to develop and validate condition-specific questionnaires with defined cut-off values for the various QoL domains being evaluated.

## Data Availability

The data that support the findings of this study are available from the corresponding author upon request.

## References

[1] Noorollahian M, Nematy M, Dolatian A, Ghesmati H, Akhlaghi S, Khademi GR (2015) Cleft lip and palate and related factors: a 10 years study in university hospitalised patients at Mashhad—Iran. Afr J Paediatr Surg 12(4):286–290 26712297 10.4103/0189-6725.172576 PMC4955481

[2] Hlongwa P, Levin J, Rispel LC (2019) Epidemiology and clinical profile of individuals with cleft lip and palate utilising specialised academic treatment centres in South Africa. PLoS ONE 14(5):e0215931 31071123 10.1371/journal.pone.0215931 PMC6508722

[3] Madaree A (2023) Epidemiology of clefts in Kwazulu Natal: comparison with systematic review analysis, similarities, and differences. J Craniofac Surg 34(1):65–69 36002921 10.1097/SCS.0000000000008957

[4] Sithole PA, Motshabi-Chakane P, Muteba MK (2022) The characteristics and perioperative outcomes of children with orofacial clefts managed at an academic hospital in Johannesburg, South Africa. BMC Pediatr 22(1):214 35440073 10.1186/s12887-022-03267-5 PMC9016974

[5] Defabianis P, Cogo C, Massa S, Romano F (2022) Oral-health-related quality of life among non-syndromic school-age children with orofacial clefts: results from a cross-sectional study in Northern Italy. Children (Basel) 9(7):1098 35884082 10.3390/children9071098 PMC9321112

[6] Kappen IF, Bittermann GK, Stock NM, Mink van der Molen AB, Breugem CC, Swanenburg de Veye HF (2019) Quality of life and patient satisfaction in adults treated for a cleft lip and palate: a qualitative analysis. The Cleft Palate-Craniofacial J 56(9):1171–80 10.1177/1055665619843410 31018676

[7] World Health Organization (1947) Constitution the World Health Organization. WHO chron 1:29–41

[8] Pinto S, Fumincelli L, Mazzo A, Caldeira S, Martins JC (2017) Comfort, well-being and quality of life: discussion of the differences and similarities among the concepts. Porto Biomed J 2(1):6–12 32258577 10.1016/j.pbj.2016.11.003 PMC6806988

[9] Lockhart E (2003) The mental health needs of children and adolescents with cleft lip and/or palate. Clin Child Psychol Psychiatry 8(1):7–16

[10] Hunt O, Burden D, Hepper P, Johnston C (2005) The psychosocial effects of cleft lip and palate: a systematic review. Eur J Orthod 27(3):274 15947228 10.1093/ejo/cji004

[11] Akinmoladun V, Ademola S, Ademola A (2017) Management of cleft lip and palate in Nigeria: a survey. Niger J Clin Pract 20(11):1355–1359 29303120 10.4103/njcp.njcp_314_16

[12] Foo P, Sampson W, Roberts R, Jamieson L, David D (2012) General health-related quality of life and oral health impact among Australians with cleft compared with population norms; age and gender differences. Cleft Palate Craniofac J 49(4):406–413 21309686 10.1597/10-126

[13] Lewis J, Patel M, Tabukumm N, Lee WC (2025) Social media depiction of cleft lip and cleft palate: instagram versus youtube shorts analysis: instagram post versus instagram reel analysis. Craniomaxillofac Trauma Reconstr 18(1):4 40271467 10.3390/cmtr18010004 PMC11995827

[14] Fayers PM, Machin D (2015) Quality of life: the assessment, analysis and reporting of patient-reported outcomes. John Wiley & Sons

[15] Haraldstad K, Wahl A, Andenæs R, Andersen JR, Andersen MH, Beisland E, Borge CR, Engebretsen E, Eisemann M, Halvorsrud L, Hanssen TA (2019) A systematic review of quality of life research in medicine and health sciences. Qual Life Res 28:2641–2650 31187410 10.1007/s11136-019-02214-9 PMC6761255

[16] Farronato G, Cannalire P, Martinelli G, Tubertini I, Giannini L, Galbiati G, Maspero C (2014) Cleft lip and/or palate. Minerva Stomatol 63(4):111–126 24705041

[17] Opriş D, Băciuţ G, Bran S, Dinu C, Armencea G, Opriş H, Mitre I, Manea A, Stoia S, Tamas T, Barbur I (2022) The quality of life after cleft lip and palate surgery. Med Pharm Rep 95(4):461 36506603 10.15386/mpr-2472 PMC9694746

[18] Berger ZE, Dalton LJ (2011) Coping with a cleft part II: factors associated with psychosocial adjustment of adolescents with a cleft lip and palate and their parents. Cleft Palate Craniofac J 48(1):82–90 20500070 10.1597/08-094

[19] Menon A, Krishnan S, Shetty V (2019) Development and application of a novel patient-reported outcome measure on QoL and facial aesthetics—a study on South Indian population. Cleft Palate Craniofac J 56(10):1340–1352 31146577 10.1177/1055665619852571

[20] Klassen AF, Tsangaris E, Forrest CR, Wong KW, Pusic AL, Cano SJ, Syed I, Dua M, Kainth S, Johnson J, Goodacre T (2012) Quality of life of children treated for cleft lip and/or palate: a systematic review. J Plast Reconstr Aesthet Surg 65(5):547–557 22118856 10.1016/j.bjps.2011.11.004

[21] Sung H, Ferlay J, Siegel RL, Laversanne M, Soerjomataram I, Jemal A, Bray F (2021) Global cancer statistics 2020: GLOBOCAN estimates of incidence and mortality worldwide for 36 cancers in 185 countries. CA Cancer J Clin 71(3):209–249 33538338 10.3322/caac.21660

[22] Post M (2014) Definitions of quality of life: what has happened and how to move on. Top Spinal Cord Inj Rehabil 20(3):167–180 25484563 10.1310/sci2003-167 PMC4257148

[23] Agbenorku P (2013) Orofacial clefts: a worldwide review of the problem. Int Sch Res Notices 2013(1):348465

[24] Stiernman M, Österlind K, Rumsey N, Becker M, Persson M (2019) Parental and health care professional views on psychosocial and educational outcomes in patients with cleft lip and/or cleft palate. Eur J Plast Surg 42:325–336

[25] Tiemens K, Nicholas D, Forrest CR (2013) Living with difference: experiences of adolescent girls with cleft lip and palate. Cleft Palate Craniofac J 50(2):27–34 10.1597/10-278 22582952

[26] Dardani C, Howe LJ, Mukhopadhyay N, Stergiakouli E, Wren Y, Humphries K, Davies A, Ho K, Weinberg SM, Marazita ML, Mangold E (2020) Cleft lip/palate and educational attainment: cause, consequence or correlation? A Mendelian randomization study. Int J Epidemiol 49(4):1282–1293 32373937 10.1093/ije/dyaa047 PMC7660147

[27] Constantin J, Wehby GL (2022) Academic outcomes of children with orofacial clefts: a review of the literature and recommendations for future research. Oral Dis 28(5):1387–1399 35080100 10.1111/odi.14137 PMC9232864

[28] Berger ZE, Dalton LJ (2009) Coping with a cleft: psychosocial adjustment of adolescents with a cleft lip and palate and their parents. Cleft Palate Craniofac J 46(4):435–443 19642774 10.1597/08-093.1

[29] Marcusson A, Paulin G, Östrup L (2002) Facial appearance in adults who had cleft lip and palate treated in childhood. Scand J Plast Reconstr Surg Hand Surg 36(1):16–23 11925823 10.1080/028443102753478327

[30] Noor SN, Musa S (2007) Assessment of patients’ level of satisfaction with cleft treatment using the Cleft Evaluation Profile. Cleft Palate Craniofac J 44(3):292–303 17477746 10.1597/05-151

[31] Al-Namankany A, Alhubaishi A (2018) Effects of cleft lip and palate on children’s psychological health: a systematic review. J Taibah Univ Med Sci 13(4):311–318 31435341 10.1016/j.jtumed.2018.04.007 PMC6694901

[32] Bos A, Prahl C (2011) Oral health–related quality of life in Dutch children with cleft lip and/or palate. Angle Orthod 81(5):865–871 21506658 10.2319/070110-365.1 PMC8916177

[33] Paganini A, Moss T, Persson M, Mark H (2021) A gender perspective on appearance-related concerns and its manifestations among persons born with unilateral cleft lip and palate. Psychol Health Med 26(6):771–778 32720821 10.1080/13548506.2020.1800055

[34] Allen PF (2003) Assessment of oral health related quality of life. Health Qual Life Outcomes 1:1–8 14514355 10.1186/1477-7525-1-40 PMC201012

[35] Payer D, Krimmel M, Reinert S, Koos B, Weise H, Weise C (2024) Oral health-related quality of life in patients with cleft lip and/or palate or Robin sequence. Journal of Orofacial Orthopedics / Fortschritte der Kieferorthopädie 85(2):98–109 35852562 10.1007/s00056-022-00414-6 PMC10879386

[36] Kramer FJ, Gruber R, Fialka F, Sinikovic B, Schliephake H (2008) Quality of life and family functioning in children with nonsyndromic orofacial clefts at preschool ages. J Craniofac Surg 19(3):580–587 18520368 10.1097/SCS.0b013e31816aaa43

[37] Sahoo AR, Dheer SS, Mahesh PC, Goyal P, Sidhu R, Deepalakshmi S (2023) A questionnaire study to assess patients with cleft lip and palate for their oral health-related quality of life. Cureus 15(5):38712 10.7759/cureus.38712 PMC10246514 37292523

[38] Peroz R, Hakelius M, Falk-Delgado A, Phua Y, Mani M (2024) Patient reported outcome following the Skoog unilateral cleft lip repair among adults-a long-term cohort study and comparison to a non-cleft population. Cleft Palate Craniofac J 61(9):1548–1558 37246371 10.1177/10556656231177139 PMC11323433

[39] Mani M, Carlsson M, Marcusson A (2010) Quality of life varies with gender and age among adults treated for unilateral cleft lip and palate. Cleft Palate Craniofac J 47(5):491–498 20180705 10.1597/08-281

[40] Eslami N, Majidi MR, Aliakbarian M, Hasanzadeh N (2013) Oral health-related quality of life in children with cleft lip and palate. J Craniofac Surg 24(4):e340–e343 23851861 10.1097/SCS.0b013e31828b743b

[41] Nolte FM, Bos A, Prahl C (2019) Quality of life among Dutch children with a cleft lip and/or cleft palate: a follow-up study. Cleft Palate Craniofac J 56(8):1065–1071 31035778 10.1177/1055665619840220

[42] Van Roy B, Groholt B, Heyerdahl S, Clench-Aas J (2010) Understanding discrepancies in parent-child reporting of emotional and behavioural problems: effects of relational and socio-demographic factors. BMC Psychiatry 10:1–2 20637090 10.1186/1471-244X-10-56 PMC2912799

